# Identification of the key mechanisms of action of Si-Ni-San in uveitis using bioinformatics and network pharmacology

**DOI:** 10.1097/MD.0000000000034615

**Published:** 2023-08-25

**Authors:** Dandan Zhang, Liu Hong, Rui Su Zhang, Qian Zhang, Jing Yao, Jiadi Wang, Ning Zhang

**Affiliations:** a Dalian Women and Children’s Medical Group, Dalian, China; b The Second Affiliated Hospital of Heilongjiang University of Chinese Medicine, Ha Er Bin Shi, China; c Heilongjiang University of Chinese Medicine, Harbin, China; d The First Affiliated Hospital of Heilongjiang University of Chinese Medicine, Harbin, China; e Banan Hospital of Chongqing Medical University, Chongqing, China.

**Keywords:** bioinformatics, network pharmacology, Si-Ni-San, uveitis

## Abstract

**Background:**

Uveitis is an eye disease with a high rate of blindness, whose pathogenesis is not completely understood. Si-Ni-San (SNS) has been used as a traditional medicine to treat uveitis in China. However, its mechanism of action remains unclear. This study explored the potential mechanisms of SNS in the treatment of uveitis through network pharmacology and bioinformatics.

**Methods:**

Using R language and Perl software, the active components and predicted targets of SNS, as well as the related gene targets of uveitis, were mined through the Traditional Chinese Medicine Systems Pharmacology, Therapeutic Target, Gene Expression Omnibus, GeneCards, and DrugBank databases. The network diagram of active components and intersection targets was constructed using Cytoscape software and the String database. The CytoNCA plug-in was used to conduct topological analysis on the network diagram and screen out the core compounds and key targets. The genes were analyzed for Gene Ontology and Kyoto Encyclopedia of Genes and Genomes enrichment. Chemoffice, Pymol, AutoDock, and Vina were used to analyze the molecular docking of key targets and core compounds of diseases through the PubChem database.

**Results:**

JUN, RELA, and MAPK may play important roles in the treatment of uveitis by SNS. Kyoto encyclopedia of genes and genomes pathway enrichment analysis showed that core genes were mainly concentrated in MAPK, toll-like receptor, tumor necrosis factor, and nucleotide oligomerization domain-like receptor signaling pathways. In addition, molecular docking results showed that the bioactive compounds (kaempferol, luteolin, naringin, and quercetin) exhibited good binding ability to JUN, RELA, and MAPK.

**Conclusion:**

Based on these findings, SNS exhibits multi-component and multi-target synergistic action in the treatment of uveitis, and its mechanism may be related to anti-inflammatory and immune regulation.

## 1. Introduction

Uveitis is a common autoimmune eye disease that could seriously harm visual health, leading to cataracts, glaucoma, keratopathy, macular edema, and even permanent vision loss.^[1,2]^ Modern medicine uses hormones and immunosuppressants as first-line treatment methods, but there are concerns regarding dependency, toxicity, and side effects, which may lead to serious complications in the eyes or the whole body; indeed, patients are prone to relapse or progression into chronic diseases following drug withdrawal.^[3]^

The clinical treatment of uveitis in China is mainly symptomatic, using a combination of traditional Chinese and Western medicine. The use of classical Chinese prescriptions often results in a better therapeutic effect and fewer adverse reactions.^[4,5]^ China has thousands of years of experience in prescribing traditional herbal medicines through traditional Chinese medicine (TCM), including for the treatment of uveitis. The use of modified Si-Ni-San (SNS) in the treatment of uveitis has achieved good clinical efficacy.^[6–9]^ SNS is composed of 4 herbs, Radix Bupleuri (Chaihu), Fructus Aurantii (Zhiqiao), paeony (Shaoyao), and licorice (Gancao) and was first referred to in a treatise on febrile diseases. It has a soothing effect on the liver and gallbladder, regulates Qi activity, and has been highly praised by physicians. However, despite its confirmed therapeutic effect as a TCM, its mechanism of action is poorly understood, the chemical composition is complex, and systematic analysis has been inadequate.

**Figure 1.**
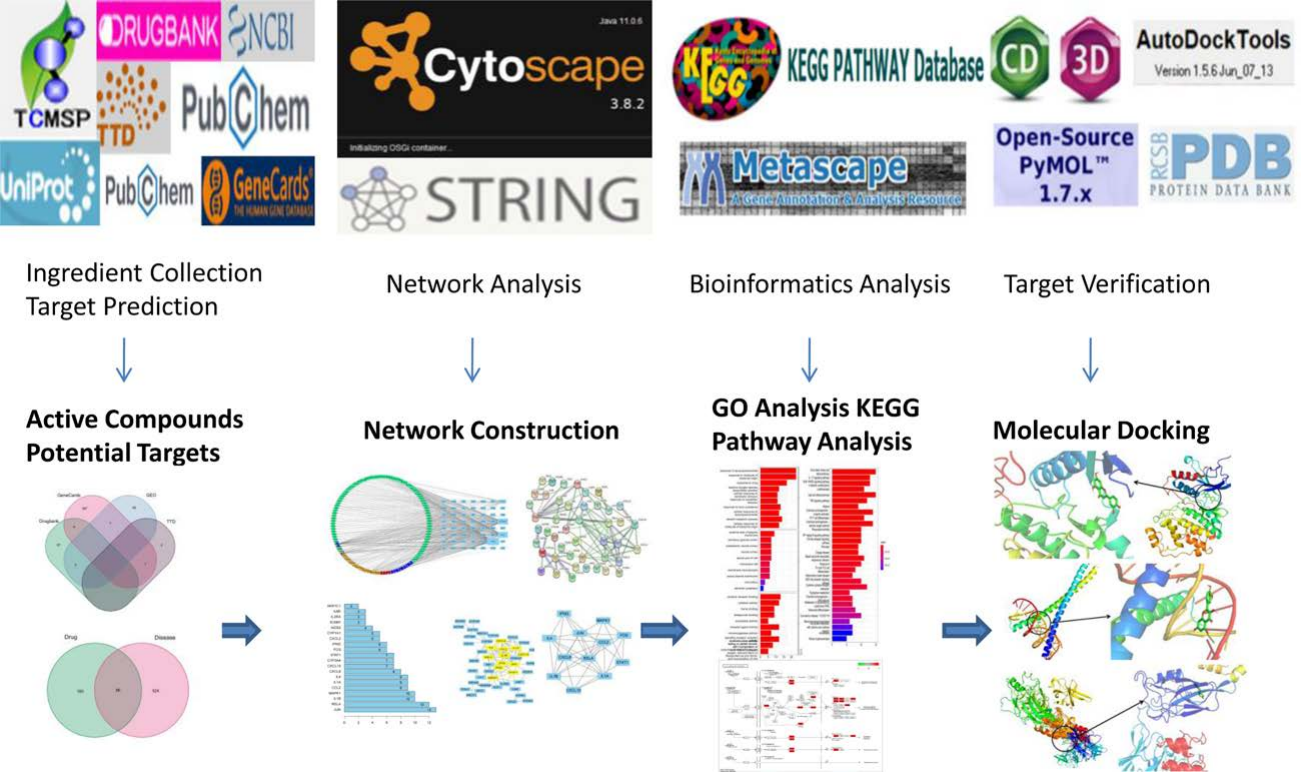
Overall flow chart of this study.

Network pharmacology systematically determines the influence of drugs on diseases based on disease–genetic or disease–drug interaction networks to characterize the multi-component, multi-target, and multi-pathway mechanisms of action of TCMs.^[10–13]^ In this study, the effect of treatment interventions with SNS on uveitis was revealed from different perspectives through data mining using network pharmacology and molecular docking technology. Potential targets and mechanisms of action of SNS in the treatment of uveitis were determined to provide a scientifically based reference for further research and clinical application of this formula. The overall flow chart of this study is shown in Figure 1.

## 2. Methods

### 2.1. Screening of active components and target proteins of SNS

The active ingredients of SNS were searched from the traditional Chinese medicine systems pharmacology (TCMSP) database (https://tcmsp-e.com/) with Radix Bupleuri, Fructus Aurantii, paeony, and licorice as keywords. The target active ingredient groups were identified with an oral bioavailability ≥ 30% and drug-like activity ≥ .18 as screening criteria. Next, the active ingredients of Chinese medicines were paired with potential targets individually, according to the TCMSP database. The UniProt database (https://uniprot.org/) was used to standardize the targets, and *Homo sapiens* was selected as the target of the active ingredients of SNS. Genetic targets of the active components of SNS were obtained using a Perl script (https://www.perl.org/).

### 2.2. Screening of targets for uveitis

Using the Therapeutic Target (https://db.idrblab.net/ttd/), DrugBank (http://www.drugbank.ca/), and GeneCards (https://www.genecards.org) databases, the keyword was set to “uveitis” and species set to “Homosapiens” to retrieve disease targets. In the Gene Expression Omnibus database (https://www.ncbi. nlm.nih.gov/geo), “uveitis” was searched as a keyword, the species was set as “Homosapiens,” and GeneChip data from the GSE66936, GSE18781, and GPL570-55999 files were used. A Perl script was used to merge data when 2 genes in different datasets were identical. Since there are batch differences in the data from different experimental environments, R (×64 4.1.0; R Foundation for Statistical Computing, Vienna, Austria) software was used for batch correction of the combined data using the SVA package, and the data were further corrected by the “limma” package with the following filter conditions: |LogFC| ≥ 1.5, *P* < .05. Uveitis genes were obtained from the GEO database. Subsequently, the “VennDiagram” package in R was used to merge disease-related genes obtained from the above databases, remove repeated targets, and integrate uveitis-related targets.

### 2.3. Construction of target protein interaction network

Venn analysis was performed on the gene targets of the effective components of SNS and the disease targets of uveitis using the “VennDiagram” package in R. Once the target SNSs in the treatment of uveitis were obtained, Cytoscape 3.8.2 software was used to construct the network maps of active ingredients and intersection targets. The intersection target was inputted into the String database (https://string-db.org/), the species was set as “Homosapiens,” the lowest interaction threshold was set as “highest confidence (.9),” and protein-protein interaction (PPI) network information was obtained. Afterward, using Cytoscape 3.8.2, the CytoNCA plug-in was used to carry out a topological analysis of the PPI network, including degree intermediary betweenness centrality value (BC), intermediate degree centrality value (DC), proximity closeness centrality (CC), feature centrality of the eigenvector (EC), local average connectivity (LAC), and network centrality scoring conditions for filtering. The screening was conducted to calculate the value corresponding to the median of nodes. Nodes higher than this value were defined as representing compounds with a significant effect and key targets.

### 2.4. Gene ontology (GO) and Kyoto encyclopedia of genes and genomes (KEGG) pathway enrichment analyses

The GO enrichment analysis of biological process (BP), cellular component, and molecular function (MF) categories was carried out using the R packages “clusterProfiler” and “enrichplot.” The KEGG pathways analysis was carried out at the same time, and the threshold was set at *P* < .05. A histogram was drawn to further reveal the mechanism of action of SNS in the treatment of uveitis.

### 2.5. Molecular docking

Small molecule ligands were downloaded from the PubChem database (https://Pubchem.Ncbi.Nim.Nih.Gov/) in the structure of the 2D mol format. Using Chemoffice software, the 2D structure was converted to a 3D structure. The Pdb format files of the 3D structures of the core target proteins were downloaded from the RCSB Protein Data Bank (http://www.rcsb.org/), and water molecules and small ligand molecules were removed using Pymol. AutoDock was used for molecular docking based on human protein. Vina and Pymol were used to plot the results with the lowest binding energy for each target.

### 2.6. Ethics and dissemination

Ethical approval was not required for this bioinformatics and network pharmacology analysis as we did not use data related to individual patients. The final report of this paper will be published in a peer-reviewed scientific journal or at conferences to provide evidence-based medical support on noninfectious uveitis, its genetics, molecular pathogenesis, and new therapeutic targets for clinicians. The dataset will be made freely available.

## 3. Results

### 3.1. Prediction of active components and related targets of SNS

A total of 144 active ingredients were screened by the TCMSP database with oral bioavailability ≥ 30% and drug-like activity ≥ .18 as screening conditions, including 92 licorice, 22 Fructus Aurantii, 13 Paeony, and 17 Radix Bupleuri. In total, 2549 targets were obtained. A total of 236 targets related to active ingredients were obtained after removing the repeated targets.

### 3.2. Prediction of uveitis disease targets and acquisition of intersection targets

**Figure 2.**
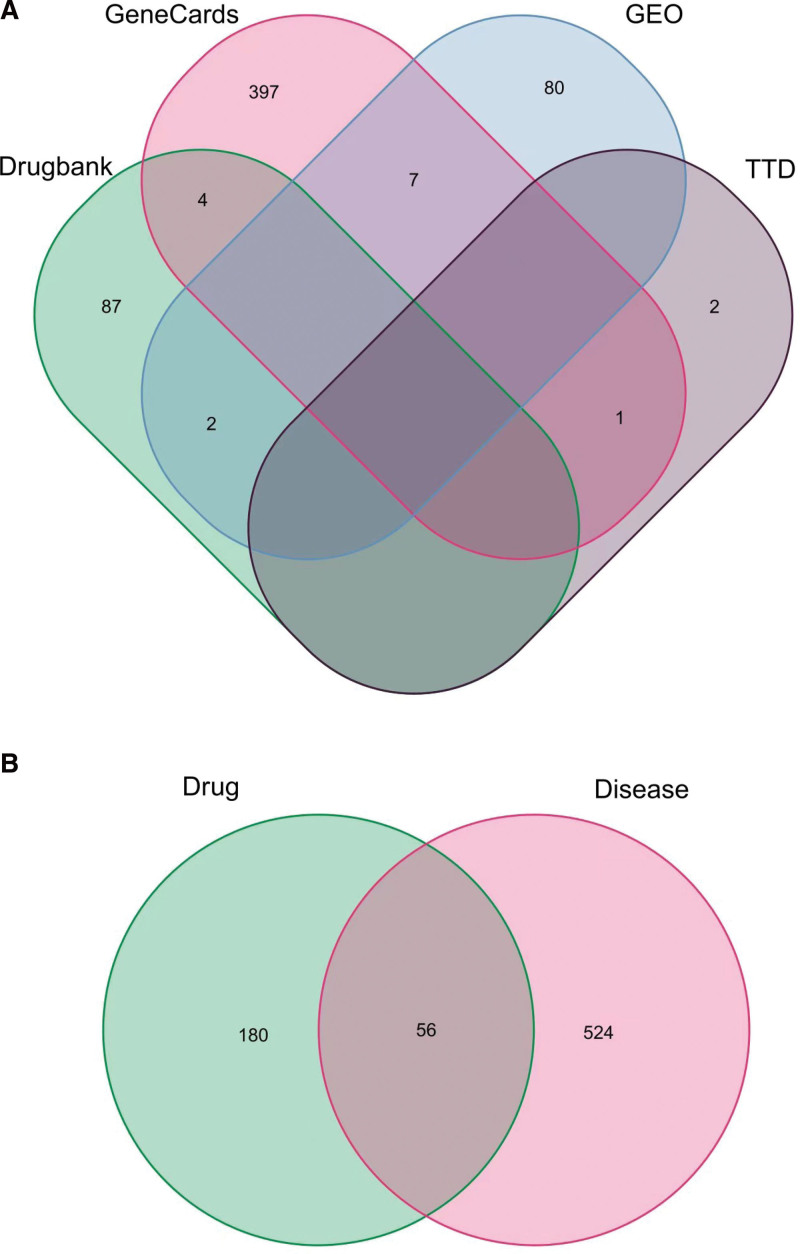
Venn diagrams of (A) uveitis targets and (B) SNS and uveitis targets. SNS = Si-Ni-San.

Through the analysis of the Gene Expression Omnibus database GSE66936, GSE18781 chip, we obtained the genetic variations of healthy people and patients with uveitis, where |LogFC| ≥ 1.5, *P* < .05 were the filtering conditions. After batch correction, a total of 89 differentially expressed genes were obtained, including 31 upregulated and 58 downregulated genes.^[14]^ In addition, uveitis-related genes were searched through Therapeutic Target, DrugBank, and GeneCards databases. A total of 580 disease-related targets were obtained by removing duplicate targets after combining genes from multiple databases (Fig. 2A). Following a Venn analysis of the genetic targets of the active components of SNS and the targets of uveitis disease, 56 SNS targets for the treatment of uveitis were obtained (Fig. 2B).

### 3.3. SNS–component–disease target regulatory network

**Figure 3.**
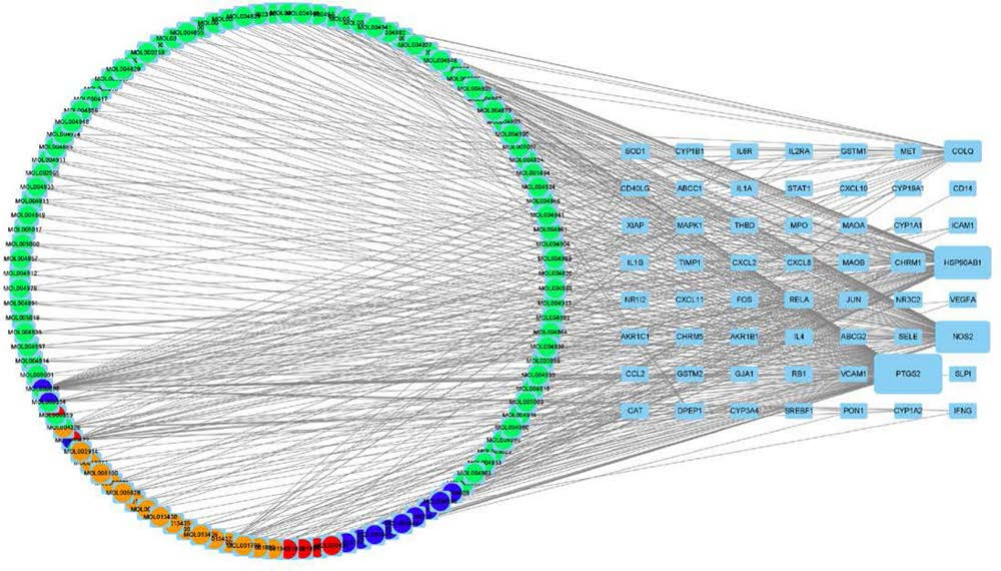
SNS–component–disease regulation network diagram. The left circle represents the TCM effective components, and the different colors represent elements from different drugs. Green represents licorice, blue represents Radix Bupleuri, red represents paeony, orange represents Fructus Aurantii, and the remaining colors represent a variety of TCMs. The grid on the right represents the target genes of SNS for the treatment of uveitis, shown in blue. The larger the area on the side of the grid, the more components are linked to the gene. SNS = Si-Ni-San, TCM = traditional Chinese medicine.

Cytoscape 3.8.2 software was used to establish the interaction network between active ingredients and targets. Nodes (170) represented the interaction between TCM, active ingredients, and targets, while edges (414) represented the interaction between active ingredients and target diseases (Fig. 3). The top 4 active ingredients in the list of targets were quercetin-MOL000098, kaempferol-MOL000422, luteolin-MOL000006, and naringin-MOL004328, which interacted with 39, 19, 15, and 11 target proteins, respectively. These may be the core active SNS ingredients in the treatment of uveitis.

### 3.4. Construction and network topology analysis of the PPI between SNS and uveitis

**Figure 4.**
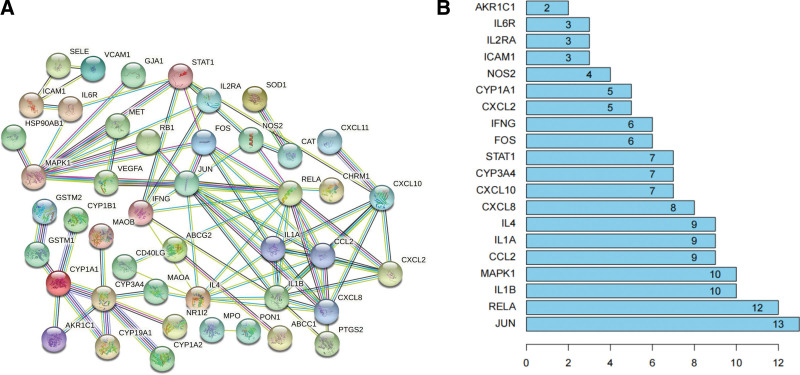
Protein-protein interaction network of uveitis.

**Figure 5.**
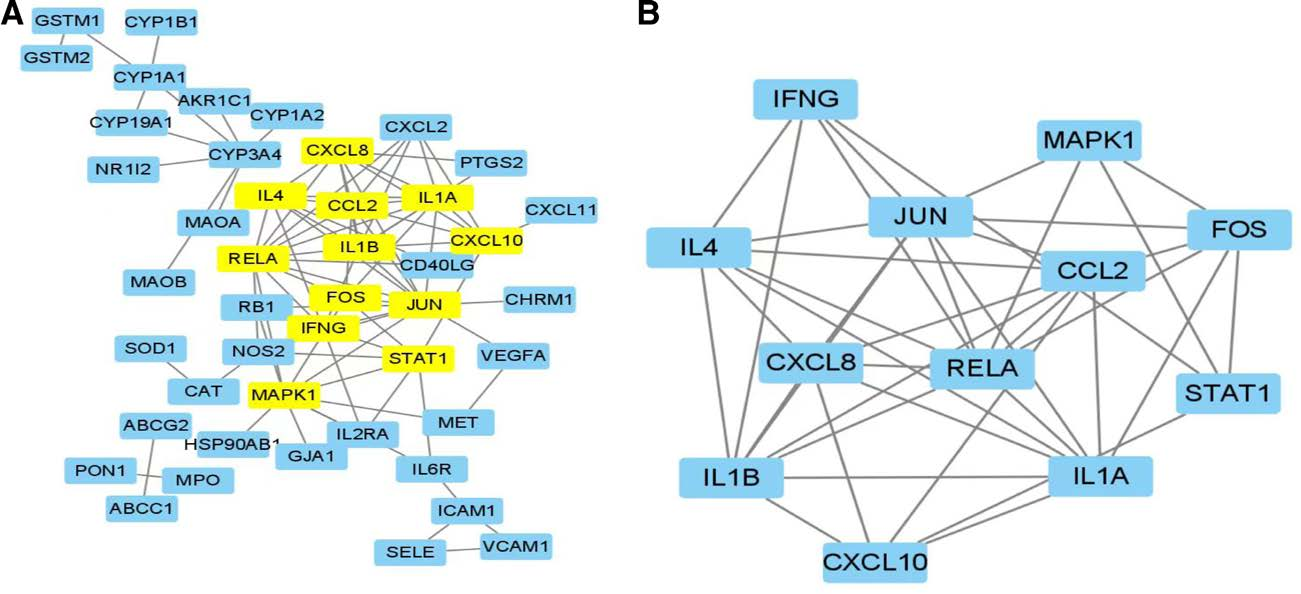
SNS–uveitis network topology analysis. (A) Filtered core network diagram, and (B) streamlined core network. SNS = Si-Ni-San.

To further study the mechanism of action of SNS in the treatment of uveitis, 56 intersection targets were analyzed using PPI networks (Fig. 4A). The most significant core gene was JUN, with a connectivity of 13, followed by RELA, interleukin (IL)-1B, MAPK1, CCL2, IL1A, IL4, CXCL8, and CXCL10 (Fig. 4B). A total of 87 edges representing the interaction between proteins were generated. The relationships and interactions between targets were identified. The constructed protein-protein interaction network was imported into Cytoscape 3.8.2, where the CytoNCA plug-in was used to calculate the score of each node, and R was used for filtering. The filtering condition was that the scores of genes in BC, DC, CC, EC, LAC, and network centrality were all greater than the median value (BC, 1.9; DC, 2; CC,.13; EC,.03; LAC,.067; NE, 2), and a list of genes satisfying the above conditions was obtained (Fig. 5A). The gene list was imported into Cytoscape 3.8.2, and a simplified core network of 12 nodes and 53 edges was obtained using the CytoNCA plug-in (Fig. 5B). The core genes were CCL2, RELA, FOS, JUN, IL1A, IL4, CXCL10, IL1B, CXCL8, STAT1, MAPK1, and IFNG. The genes with high connectivity to the active ingredients were selected for molecular docking analysis.

### 3.5. GO enrichment and KEGG pathway enrichment results

**Figure 6.**
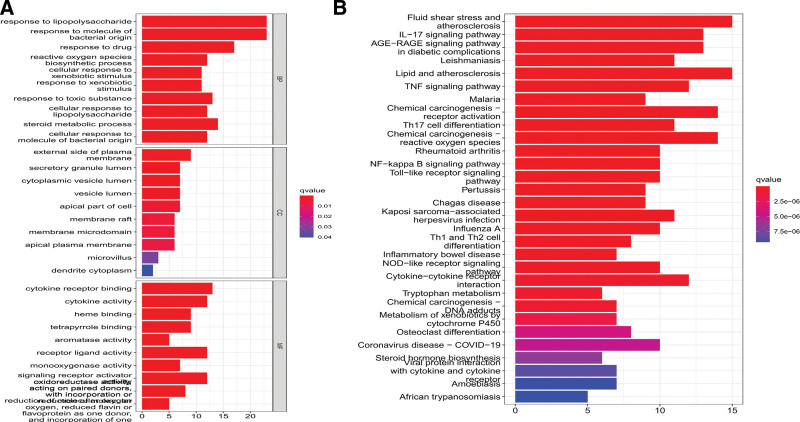
Gene enrichment at the intersection of SNS–uveitis. (A) GO enrichment results, and (B) KEGG enrichment results. GO = gene ontology, KEGG = Kyoto encyclopedia of genes and genomes. SNS = Si-Ni-San.

A total of 1478 functions were obtained, including 1339 for BP, 11 for CC, and 126 for MF. The top 8 functions were screened and plotted (Fig. 6A). The BPs mainly involved responses to lipopolysaccharides (LPSs), molecules of bacterial origin, reactive oxygen species, and biosynthetic BPs, such as the regulation of the nasal metabolic process. The CCs included the external side of the plasma membrane, cytoplasmic vesicle lumen, secretory granule lumen, membrane raft, and membrane microdomain. The MFs were mainly enriched in cytokine receptor binding, cytokine activity, aromatase activity, receptor-ligand activity, monooxygenase activity, signaling receptor activator activity, and oxidoreductase activity.

**Figure 7.**
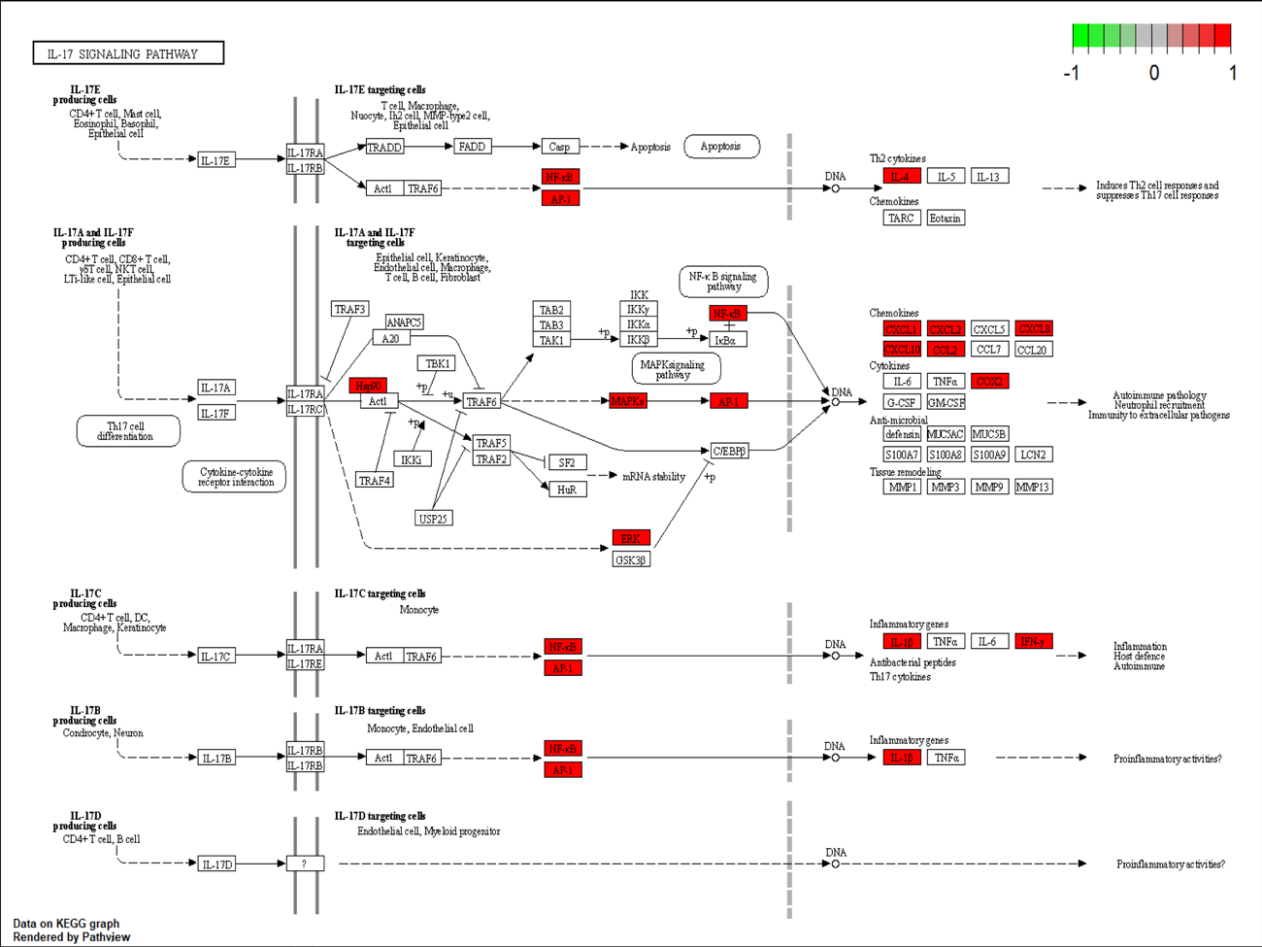
IL-17 signaling pathway diagram.

A total of 122 pathways were obtained through the KEGG pathway enrichment analysis, and the first 30 pathways were screened to construct a chart (Fig. 6B). Several signaling pathways were closely associated with uveitis, including Toll-like receptor, Il-17, MAPK, TNF, and NOD-like receptor signaling pathways, suggesting that SNS can act on uveitis through multiple channels. Using the IL-17 signaling pathway as an example, the potential targets and mechanisms of action of SNS in the treatment of uveitis were mapped (Fig. 7).

### 3.6. Molecular docking verification results

**Figure 8.**
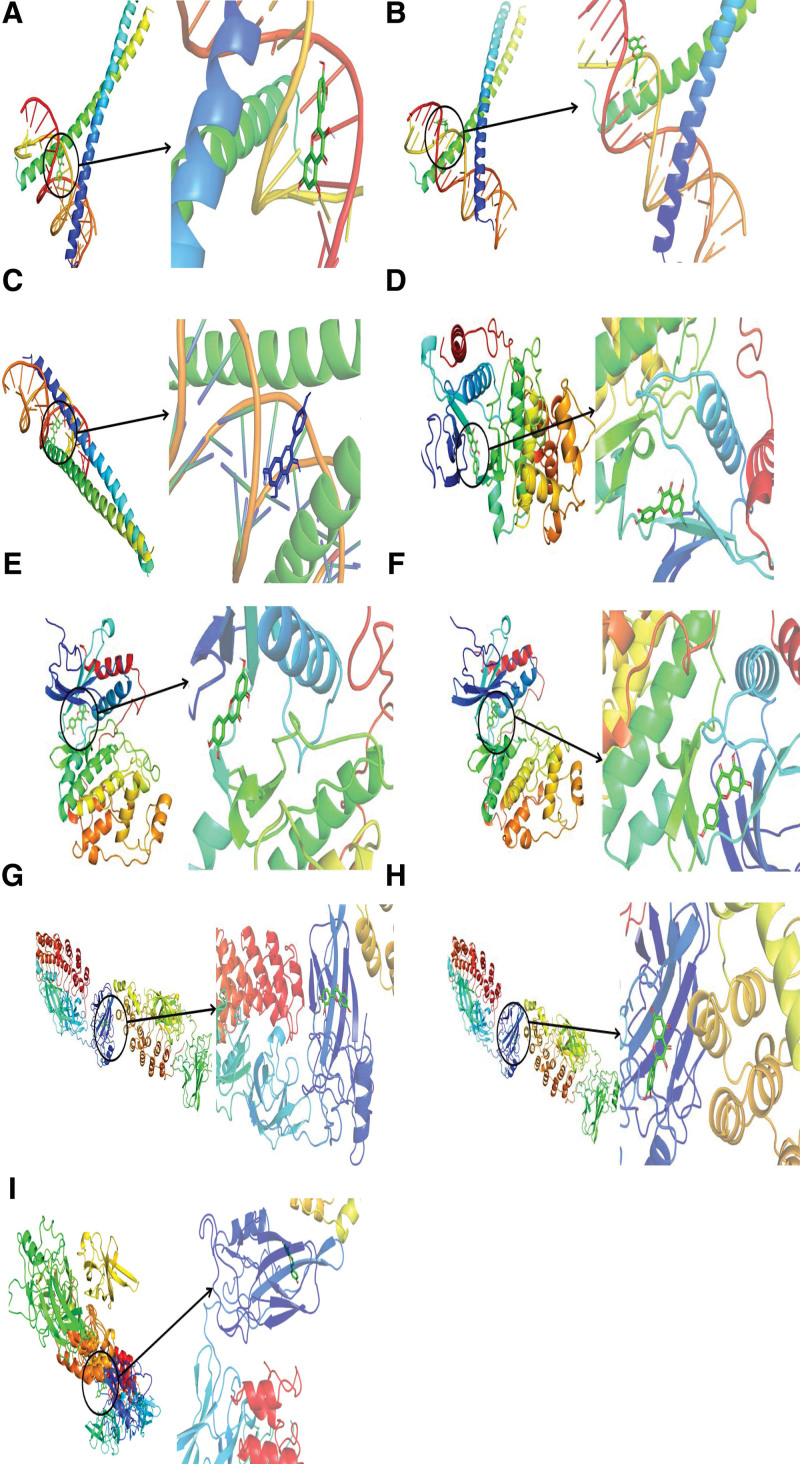
Molecular docking results: (A) JUN–quercetin, (B) JUN–luteolin, (C) JUN–kaempferol, (D) MAPK1–luteolin, (E) MAPK1–naringenin, (F) MAPK1–quercetin, (G) NFKB3–quercetin, (H) NFKB3–naringenin, and (I) NFKB3–luteolin.

The core genes obtained from the network topology analysis were sorted by degree value, and the first 3 key targets (JUN, RELA, and MAPK1) were selected for analysis of their molecular interconnection with 4 key pharmacodynamic components (quercetin, kaempferol, luteolin, and naringin) for SNS treatment of uveitis. The lower the energy of the stable conformation of ligand and receptor binding, the more likely the interaction will occur. In this study, the molecular docking results showed that the binding energy of JUN and quercetin was − 8.6 kcal/mol, that of JUN and luteolin was − 8.8 kcal/mol, and that of JUN and kaempferol was − 8.7 kcal/mol. The binding energy of MAPK1 to quercetin was − 8.4 kcal/mol, that of MAPK1 to luteolin was − 8.6 kcal/mol, and that of MAPK1 to naringin was − 8.4 kcal/mol. The binding energy of NFKB3 to luteolin was − 7.7 kcal/mol, that of NFKB3 to quercetin was − 7.7 kcal/mol, and that of NFKB3 to naringin was − 7.4 kcal/mol (Fig. 8).

## 4. Discussion

SNS is a Shanghan-lun (a TCM archive) prescription composed of 4 medicinal ingredients that elicit an effective treatment response. SNS functions in immune regulation, sedation, hypnosis, liver protection, and lipid-lowering, anti-inflammatory, and antidepression responses and can be used to treat uveitis.^[15–18]^ In the present study, TCMSP was used to obtain 144 active components of SNS, and 236 potential therapeutic targets were identified through network pharmacology. Furthermore, 56 potential genes for SNS treatment of uveitis were obtained. There were 170 nodes and 414 edges obtained in the PPI. In the GO analysis, 1339 BPs, 126 MFs, and 11 CCs were enriched, and 122 related pathways were enriched in the KEGG analysis. The molecular docking of 3 targets and 5 active ingredients provided an in-depth analysis of genes and drug components, further confirming the anti-inflammatory and immunomodulatory effects of SNS.

By analyzing the PPI network of SNS in the treatment of uveitis, we found that the core therapeutic genes were *CCL2, RELA, FOS, JUN, IL1A, IL4, CXCL10, IL1B, CXCL8, STAT1, MAPK1*, and *IFNG*. These genes were involved in BPs such as LPS reactions, responses to bacteria-derived molecules, reactive oxygen species biosynthesis, and regulation of steroid metabolism. In addition, studies have shown that MAPK1, RELA, and JUN can effectively control the progression of uveitis.^[7,19,20]^

Kaempferol, luteolin, naringin, quercetin, and other main components of TCM docked with the molecule also exhibited anti-inflammatory and immunosuppressive effects. Many herbs in SNS contain kaempferol, which has anti-inflammatory, antioxidant, neuroprotective, and other pharmacological effects.^[21–23]^ Luteolin has neuroprotective effects by reducing oxidative stress and antioxidant activity. Luteolin also inhibits the LPS response, inhibits the activation of JNK, P38, ERk, NF-κB, and STAT3, and modulates transcription factors such as STAT3, NF-κB, and AP-1 to achieve anti-inflammatory effects.^[24–26]^ Luteolin enhances the kaempferol-inhibited expression of drug-metabolizing enzymes, and co-treatment with kaempferol and luteolin was previously found to increase the cellular levels of kaempferol without affecting the levels of luteolin.^[27]^ Quercetin can inhibit the activation of P38, ERK1/2, and NF-κB; reduce the expression of cyclooxygenase and lipoxygenase; maintain the stability of mast cells; reduce the production of IL-1β, IL-6, and TNF-α; inhibit Lyn/PLCγ/IP3R-Ca^2+^, Lyn/ERK1/2, and Lyn/NF-κB signaling pathways; and achieve anti-inflammatory effects.^[28–30]^ Naringin can reduce the release of IL-1β, IL-6, and IL-18 and inhibit the high expression of NF-κB-P65. Inflammation is inhibited through PI3K/Akt and MAPK/ERK signaling pathways.^[31,32]^

According to the KEGG enrichment analysis, the important pathways involving immune regulation in the SNS treatment of uveitis include the TLR and NOD-like receptor signaling pathways and Th17 cell differentiation. Pathways involved in the inflammatory response include IL-17, TNF, MAPK, and NF-κB signaling pathways, the AGE-RAGE signaling pathway with diabetic complications, and cytokine–cytokine receptor interaction. Kaempferol regulates signaling pathways associated with TNF, Toll-like receptor, NF-κB, NOD-like receptor, rheumatoid arthritis, IL-17, MAPK, lipids, and atherosclerosis.^[33–36]^ Luteolin regulates signaling pathways associated with TNF, lipids, atherosclerosis, NF-κB, Toll-like receptors, and Nod-like receptors.^[37–39]^ Quercetin regulates signaling pathways associated with NOD-like receptors, MAPK, and TLR.^[40–42]^ Naringin regulates signaling pathways associated with NOD-like receptors, MAPK, and TLR.^[43,44]^ According to our PPI network and KEGG enrichment analysis, kaempferol was associated with the most regulatory pathways and the most potential targets, indicating that the pharmacological results of the SNS network are consistent with the clinical treatment results of uveitis.

The inflammatory immune response is an important factor in uveitis. According to GO and KEGG enrichment analyses, the potential target of the main components of SNS in the treatment of uveitis is inflammatory immune-related pathways. Among them, the MAPK signaling pathway, which was associated with abundant targets in the current study, is an important target pathway. Studies have shown that the T-cell receptor signaling pathway is involved in immune defense through the p38 MAPK signaling pathway.^[45,46]^ In addition, TLR enrichment and IL-17 signaling pathways have been extensively studied^[19,47–49]^ TLR protein expression was activated, and CXCL1 and CXCL2 proteins were significantly increased in both time- and dose-dependent manners. The enhanced activity of the IL-17 pathway is associated with the proliferation and pathogenicity of Th17 cells, and evidence from animal models has suggested that the development of pathogenic Th17 cells is responsible for experimental autoimmune uveitis.^[47]^ The helper T cells of IL-17, specifically Th1 and Th17 cells, can promote the proliferation of Tregs and T cells, which are most abundant in MAPK and TNF signaling pathways. They regulate the immune response and the expression of inflammatory factors caused by uveitis. The main components observed in the present study also intervene in inflammatory and immune-related pathways.^[25,43,50–53]^ Kaempferol anti-inflammatory effect is mediated by the inhibition of MAPK-related extracellular signal-regulated kinase and the P38 signaling pathways. Kaempferol inhibits TNF-α-induced MAPK activation without affecting TNF-α receptor expression. Luteolin inhibits IL-1β, IL-17, and TNF-α and regulates NF-κB, JAK-STAT, and TLR signaling pathways. It also inhibits the activation of NLRP3 inflammasomes and promotes the differentiation of macrophages into the M2 phenotype to achieve the anti-inflammatory effect. Quercetin ameliorated neuro-inflammation in mice by modulating the NLRP3 pathway in NOD-like receptor signaling, which was demonstrated by reducing IL-1β and IL-18 levels.^[52]^ Naringin inhibits NF-κB activation and the NOD-like receptor family in NLRP3 inflammasomes.^[53]^ The key targets and pathways screened in the current study are potential targets and pathways for the therapeutic efficacy of uveitis. We also confirmed the characteristics of multiple components acting on different targets through various channels to provide auxiliary treatment ideas for SNS in the clinical treatment of uveitis.

## 5. Conclusion

In summary, we explored the potential mechanisms of SNS in the treatment of uveitis based on network pharmacology and molecular docking. The potential functional components kaempferol, luteolin, quercetin, and naringin were identified. Cumulatively, the components may regulate BPs such as immune and inflammatory responses and atherosclerosis through molecular docking targets and enriched pathways. These experiments provide a reference of the therapeutic effects of SNS on uveitis in the absence of clinical trials. In future research, we will provide biological experimental evidence for the therapeutic effects of SNS on uveitis according to the results of this study.

## Acknowledgments

We would like to thank Editage (www.editage.cn) for English language editing.

## Authors contributions

**Conceptualization:** Ning Zhang.

**Data curation:** Ning Zhang, Ruisu Zhang, Qian Zhang, Jing Yao.

**Formal analysis:** Ning Zhang.

**Methodology:** Dandan Zhang, Liu Hong, Ruisu Zhang.

**Software:** Dandan Zhang, Qian Zhang.

**Writing – original draft:** Ning Zhang, Dandan Zhang, Liu Hong.

**Writing – review & editing:** Ning Zhang, Dandan Zhang, Jiadi Wang.
